# A New Methodology for Evaluation of Nematode Viability

**DOI:** 10.1155/2015/879263

**Published:** 2015-03-19

**Authors:** Sebastião Rodrigo Ferreira, Tiago Antônio Oliveira Mendes, Lilian Lacerda Bueno, Jackson Victor de Araújo, Daniella Castanheira Bartholomeu, Ricardo Toshio Fujiwara

**Affiliations:** ^1^Departamento de Parasitologia, Universidade Federal de Minas Gerais, Avenida Antônio Carlos 6627, Pampulha, 31270-901 Belo Horizonte, MG, Brazil; ^2^Departamento de Medicina Veterinária, Universidade Federal de Viçosa, Avenida P.H. Rolfs, s/n, 36570-000 Viçosa, MG, Brazil

## Abstract

Nematodes infections are responsible for debilitating conditions and economic losses in domestic animals as well as livestock and are considered an important public health problem due to the high prevalence in humans. The nematode resistance for drugs has been reported for livestock, highlighting the importance for development of new anthelmintic compounds. The aim of the current study was to apply and compare fluorimetric techniques using Sytox and propidium iodide for evaluating the viability of *C. elegans* larvae after treatment with anthelmintic drugs. These fluorescent markers were efficient to stain larvae treated with ivermectin and albendazole sulfoxide. We observed that densitometric values were proportional to the concentration of dead larvae stained with both markers. Furthermore, data on motility test presented an inverse correlation with fluorimetric data when ivermectin was used. Our results showed that lower concentrations of drugs were effective to interfere in the processes of cellular transport while higher drugs concentrations were necessary in order to result in any damage to cell integrity. The methodology described in this work might be useful for studies that aim to evaluate the viability of nematodes, particularly for testing of new anthelminthic compounds using an easy, economic, reproducible, and no time-consuming technique.

## 1. Introduction

Intestinal nematode infections represent a debilitating threat for livestock and domestic animals worldwide as it may cause reduction in growth and weight gain and, depending on the severity of infection, it can lead to animal death. Moreover, production costs associated with treatment and control measures of parasitic infections can be very expensive [1]. In humans, the intestinal nematode infections (e.g., soil-transmitted helminthes) are highly prevalent and affect at least 1 billion people worldwide [2]. The control of nematodes is generally performed by the use of commercially available chemical compounds such as avermectins, benzimidazoles, and imidazothiazoles [3]. Despite the availability of several drugs for nematodes control, resistance to the main drugs has been extensively reported for livestock, highlighting the need to found new anthelmintic compounds [4, 5].

*Caenorhabditis elegans*, a free-living bacteriovorus nematode, has been used as a good model for research new novel anthelmintics [6]. This nematode has been valuable in basic research on anthelmintic pharmacology of human and agricultural parasites [7] as well as at understanding the mechanisms of resistance to anthelmintics [8], mainly due to the phylogenetic relationship to other parasitic helminthes. Several characteristics make* C. elegans* a good model, such as cycle fast, easy laboratory maintenance, knowledge of its genome, and phylogenetic proximity to other nematodes [9–11]. Currently, a number of techniques are available for measuring* C. elegans* viability after treatment with known drugs or candidates, such as larval development assays (ADLs, reproduction responses), or by the assessment of motility and colorimetric assays [12–15]. However, some of these techniques are time consuming and it can be subjective. According to Dickson and Gagnon [16], the discovery of new bioactive molecules is a long and expensive work, requiring investment of an average of 10 to 20 years and more than 200 million of dollars. Thus, the development of no subjective, economic, reproducible, and no time-consuming techniques would be useful.

Techniques that employ fluorescent markers such as propidium iodide and Sytox have been routinely used to measure cellular viability of mammalian cell [17]. Propidium iodide and Sytox also demonstrated to be efficient at staining nematode larvae [18, 19]. Sytox intercalates into nucleic acid and it is not able to passively cross the plasma membrane of viable cells [20], propidium iodide, which is also an intercalator to nucleic acid in cell death and, nevertheless, is also capable of going through intact cell membranes; however, it is expelled by viable cells [21]. Therefore, both markers stain nonviable cells. In this study, we applied and compared a feasible method, using the fluorescent markers propidium iodide and Sytox to evaluate the viability of* C. elegans* larvae after drug treatments.

## 2. Methodology

### 2.1. *C. elegans* L_3_ Production

The strain of* C. elegans* was kindly provided by Professor Carlos Eduardo Winter (Universidade de São Paulo (USP)). L_3_ larvae of* C. elegans* were grown on NGM plates 8P according to the methodology previously described [22, 23]. After seven days of culture in BOD incubator at 20°C, the plates were washed with M9 medium [22] and filtered through three sieves with pores of 40 *μ*m, 30 *μ*m, and 20 *μ*m. L_3_ larvae retained in the 20 *μ*m strainer were collected by backwashing. The obtained larvae were washed by centrifugation at 700 g for 4 minutes, followed by two washes with M9 medium. Larvae average size was 527 *μ* (*σ* 3.4) long by 23.3 *μ* in diameter (*σ* 1,9) [24].

### 2.2. Drug Tests with Fluorescent Markers

In order to evaluate the proportion between the number of larvae (L_3_) and the fluorescence signal, initial concentration of 2000 L_3_ larvae per well diluted in M9 medium was used for fluorimetric tests followed by 1 : 2 serial dilution of L_3_ larvae per well. Briefly, 100 *μ*L of larvae suspension was added to each well in a 96-well microplate, followed by immediate addition of 100 *μ*L of the tested drugs solution (Albendazole sulfoxide, ivermectin, Sigma-Aldrich). Stock solutions (20 mM) for all drugs were prepared in M9 solution supplemented with 0.05% DMSO (Vetec, BR) and stored at −20°C protected from light. For albendazole sulfoxide, several concentrations (4000, 3000, 2000, 1000, 500, 100, 10, 1, 0.1, and 0.01 *μ*M) were tested. Ivermectin was tested at these concentrations: 1000, 500, 100, 10, 1, 0.1, and 0.01 *μ*M. The negative control was M9 solution with 0.05% DMSO, and methanol (CH_3_OH) was used as positive control at the following dilutions 50, 25, 5, 0.5, 0.05, and 0.005% [25, 26]. Quadruplicates were performed for each drug concentration and for controls. Incubation of microplates with different drugs was performed in a BOD (incubator) at 20°C.

### 2.3. Staining with Propidium Iodide and Sytox

After 48 and 72 hours of incubation with the drugs, propidium iodide (Invitrogen, USA) and Sytox (Invitrogen, USA) were added to the microplates markers at a final concentration of 20 *μ*M and 1 *μ*M per well, respectively [18, 19]. Microplates were incubated for 15 minutes at room temperature in a horizontal shaker at 120 rpm followed by reading at LAS ImageQuant^tm^ GE 4000 with excitation in white light and emission at 605 nm for propidium iodide and 575 nm for Sytox. Densitometric analyses of the images were performed using the software GE Image Quant TL 8.1. Images were taken at microscope (Leica DM500) 100x magnification: excitation at 510–560 nm and emission at 590 nm for propidium iodide, excitation at 450–490 nm and emission at 535 nm for Sytox using a capture system (Canon EOS 600D).

### 2.4. Motility Test

*C. elegans* L_3_ were resuspended in M9, and then approximately 1000 larvae in 100 *μ*L of suspension were added to each well in a 96-well microplate. Tested drugs were then added at the same concentrations described in fluorimetric methods. Microplates containing drugs and larvae were stored in BOD incubator at 20°C. After 48 and 72 hours, 10 *μ*L of solution containing ca. 100 larvae was removed from each well for analysis and quantification of paralyzed larvae number using an optical microscope at 100x magnification. Larvae were considered paralyzed when presenting with straight body and absence of any motility [27].

### 2.5. Statistical Analyses

Data from densitometry and motility assays were tested by analysis of variance (ANOVA) and linear and nonlinear regression using the statistical program GraphPad Prism 5.0. The model was considered adequate to the data when *r* ^2^ was above 0.8 for nonlinear regression and 0.95 for linear regression. Comparison of groups was performed using normality test of Kolmogorov-Smirnov, followed by two-way ANOVA; comparison of means was tested using Bonferroni correction test for multiple hypothesis. Correlation analysis was performed using Spearman rank correlation. Nonlinear regression analysis was used to calculate the IC_50_ value.

## 3. Results

**Figure 1:**
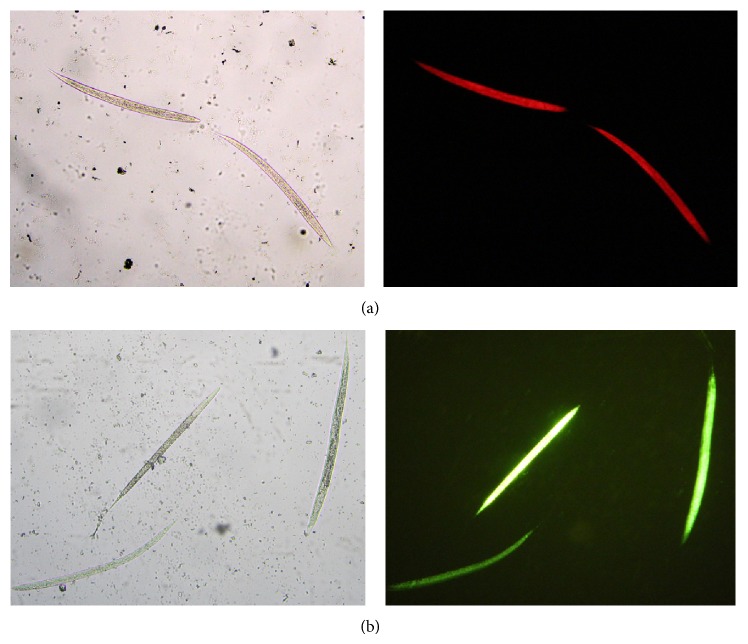
Dead* C. elegans* larvae (L_3_), bright field images on the left, and fluorescence images of same larvae on the right, propidium iodide (a) and Sytox (b).

**Figure 2:**
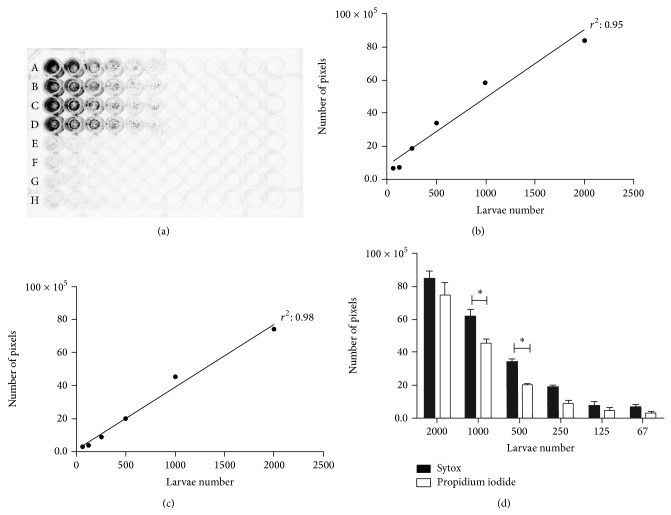
Analysis of viability using fluorimetric markers. (a) Microplate with serial dilution of* C. elegans *L_3_ (first point 2,000 to 62). Rows A–D dead larvae; E–H represent viable (control) larvae. Correlation between the number of dead larvae with 50% methanol and the fluorescence intensity of the pixels with Sytox (b) and propidium Iodide (c). Comparison of densitometry (number of pixels) between Sytox and propidium iodide markers (d). Significant differences (*P* < 0.05) were only detected when 1000 and 500 larvae were tested.

Both Sytox and propidium iodide were effective for staining larvae of* C. elegans* previously killed by treatment with 50% methanol (Figure 1), presenting a clear differentiation to viable larvae, which did not present any fluorescence (Supplementary Figure S1 in Supplementary Material available online at http://dx.doi.org/10.1155/2015/879263). When suspension of dead larvae was serially diluted and stained with both fluorescent markers (Figure 2(a)), the observed densitometric values were proportional to the concentration of dead larvae stained with Sytox (Figure 2(b)) and propidium iodide (Figure 2(c)). The comparison of the densitometric values obtained by staining with Sytox and propidium iodide demonstrates that significant differences were observed only at high number of larvae (500 and 1000 larvae) (*P* < 0.05) (Figure 2(d)).

**Figure 3:**
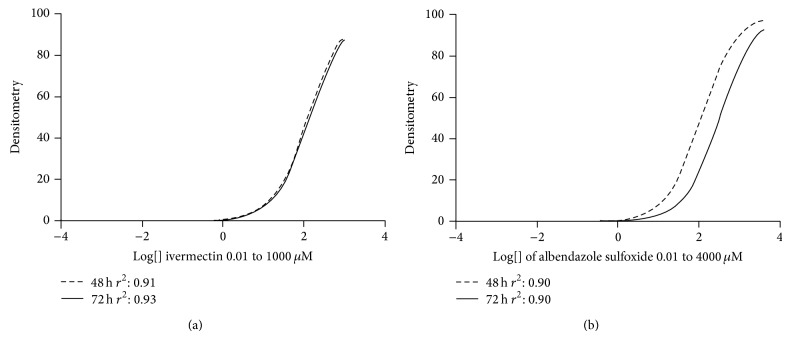
*C. elegans *larvae (L_3_) treated with ivermectin (a) at concentrations 0.01 to 1000 *μ*M (identical curves) and albendazole sulfoxide (b) at concentrations 0.01 to 4000 *μ*M, stained with propidium iodide. The larvae were incubated with the drugs for 48 and 72 hours in all treatment.

**Figure 4:**
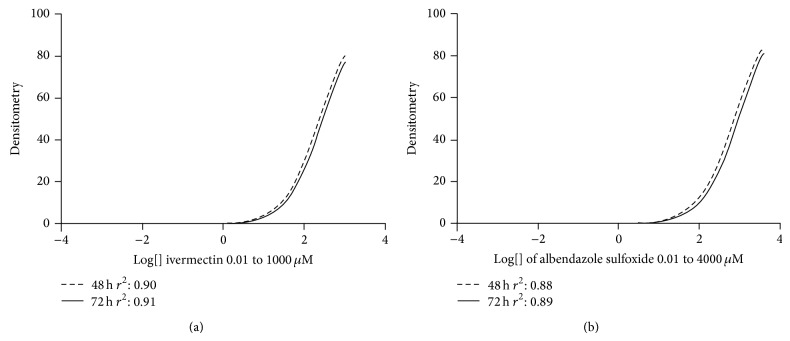
*C. elegans *larvae (L_3_) treated with ivermectin (a) at concentrations 0.01 to 1000 *μ*M and albendazole sulfoxide (b) at concentrations 0.01 to 4000 *μ*M, stained with Sytox. The larvae were incubated with the drugs for 48 and 72 hours in all treatment.

**Figure 5:**
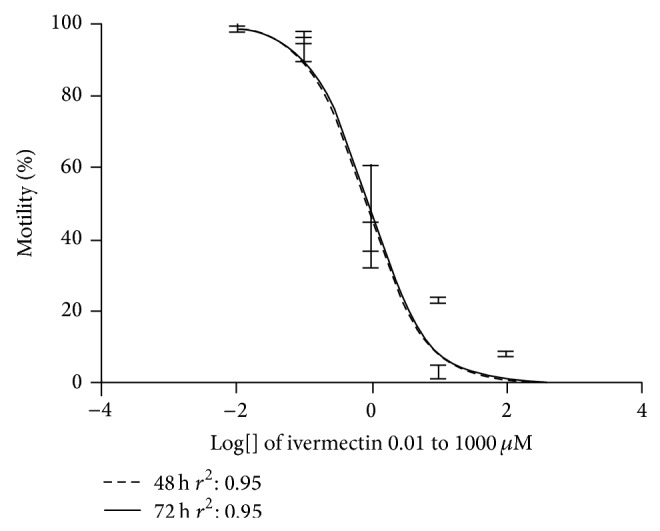
Motility analysis of* C. elegans* larvae (L_3_) treated with the ivermectin. The larvae were incubated with the drugs for 48 and 72 hours in all treatment.

**Figure 6:**
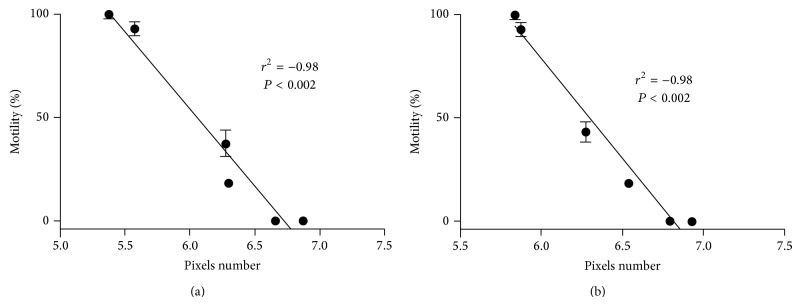
Correlation of paralysis and number of pixels from fluorimetric assays. (a) Propidium iodide and (b) Sytox. Statistical significance was determined by Spearman rank correlation.

Figure 3 shows the densitometric data of the larvae treated with albendazole sulfoxide and ivermectin and stained with propidium iodide. Our data suggest that the higher concentrations of drugs might affect the mechanisms used to expel the marker, once propidium iodide labels the nucleic acids when it remains within the cell. The densitometric data of larvae treated with the same drugs and stained with Sytox suggests that higher concentrations of drugs were able to induce damage in the cellular integrity of the larvae, because this marker is not able to overcome intact membranes (Figure 4). Concerning the ivermectin treatment, we observed that at the highest concentrations all larvae were practically inert (Figure 5). For instance, at concentration of 1000 uM, 100% of paralysis was observed when compared to control group (*P* < 0.05). A negative correlation (Spearman rank correlation, *P* < 0.05) was observed when paralysis and densitometry data were compared (Figure 6). The albendazole sulfoxide treatment induced a slightly reduction of the movement; however, body straight shapes and absence movement were not observed; thus, motility assay could not be properly performed (data not shown). The IC_50_ dates are demonstrated at Table 1, where different IC_50_ were observed. IC_50_ data for albendazole sulfoxide motility test were not included once the larvae did not fit the criteria used for impairment of motility. Larvae were considered paralyzed when presenting straight body and absence of any motility; the treatment with albendazole sulfoxide induced reduction of the larval movement but body straight shapes and absence of movement were not observed.

## 4. Discussion

In this study, we aimed to establish a methodology based on staining dead cells with the fluorescent markers Sytox and propidium iodide to evaluate the viability of* C. elegans* and its further use on testing of novel anthelmintic compounds for control of human and animal nematode infections.

During the standardization of the technique, the densitometric values observed for positive controls (larvae treated with 50% methanol, a concentration known to kill the larvae [25, 26]) were indeed proportional to the number of dead larvae, demonstrating the ideal number of larvae for the subsequent testing trials. Different number of larvae has been reported previously in the testing and standardization of new methodologies for several nematodes with a variety of IC_50_ has been observed [14, 28, 29]. According to Smith et al. [30], such discrepancies may be attributed to differences in worm strain susceptibility against the toxic agent or variation in the methods employed in the analysis or effective drug concentration.

Once the standardization of number of larvae was performed, the staining methodologies were further tested with a representative compound of each main class of available drugs currently used. When ivermectin and albendazole sulfoxide were used to induce worm death, we observed that both fluorimetric markers were efficient to stain dead larvae. Considering the mechanism of action of propidium iodide and Sytox [20, 21], our results allowed us to infer that treatment of larvae with ivermectin and albendazole sulfoxide may induce damage on the mechanism of exclusion of exogenous substances and/or affect the integrity of cell membranes. Indeed, the association of ivermectin association with glutamate-gated chloride channels (GluCl) results in the influx of chloride ion and consequent hyperpolarization of nematode muscle, culminating in the disarray on the locomotion mechanism and “pumping” of nutrients through the pharynx, promoting a flaccid paralysis and death of organism [31]. The albendazole sulfoxide is tubulin ligands with high and selective affinity for *β*-tubulin molecules, disturbing the microtubules polymerization and consequently preventing transport system mediated by microtubules [29].

Our results demonstrated that propidium iodide detected a smaller IC_50_ for ivermectin and albendazole sulfoxide, which is probably related to the cellular mechanism of influx or efflux responsible for excretion of several analytes including propidium iodide, suggesting that a relative lower drug concentration is sufficient to interfere with the mechanism of transport and the efflux of propidium iodide [29, 32, 33]. The ivermectin IC_50_ obtained with the motility assay was considerably lower than the IC_50_ obtained for fluorimetric assay, suggesting that a lower drug concentration is sufficient to impair the locomotion and disposition of worm body muscle as already described [27]. On the other hand, in order to disturb the mechanisms of exclusion of exogenous molecules or induce damage of cell membrane, higher concentrations of the drugs are required, and it is known that used drugs in the present work can damage transport protein and cell membrane [32, 33]. Interestingly, the negative correlation between motility data and number of pixels in the fluorimetric assay indicates that ivermectin induces an efficient paralysis.

Taken together, our results show that fluorimetric microplate reading tests using propidium iodide and Sytox were efficient for larvae viability analysis after treatment with ivermectin and albendazole sulfoxide. This provides a simple and viable analysis technique to probe viability nematodes using* C. elegans* as a model, likely that is easy, less subjective, economic, reproducible, and no time consuming.

## Supplementary Material

The Supplementary Material demonstrate that fluorimetric markers employed in the study does not stain viable live larvae (Supplementary Figure 1) and absence of autofluorescence emitted by the larvae, demonstrate by the lack of fluorescence when dead larvae (treated with methanol) were not stained with Sytox and Propidium Iodide. Os marcadores utilizados não foram eficazes em marcar larvas viáveis (não tratadas). A fluorescência analisada referente às larvas não tratadas com metanol não apresentava proporcionalidade, de acordo os parâmetros estabelecidos.

## Figures and Tables

**Table 1:** IC_50_ drugs: ivermectin and albendazole sulfoxide, using different fluorimetric markers and motility test (means of times 48 and 72 hours). Different letters in the lines demonstrate that the means are different (*P* < 0.05) according to Tukey test and *t*-test.

Drug	IC_50_ P. iodide	IC_50_ Sytox	IC_50_ motility
Ivermectin	132.4 *µ*M ± 5.3^a^	261.4 *µ*M ± 23.7^b^	0.87 *µ*M ± 0.05^c^
Albendazole sulfoxide	214.9 *µ*M ± 100.2^a^	874.5 *µ*M ± 67.50^b^	—
