# Supplementary figures and images for: A New Methodology for Evaluation of Nematode Viability
Source: Biomed Res Int. 2015 Mar 19;2015:879263. doi: 10.1155/2015/879263 (PMC4383492; doi:10.1155/2015/879263)

**A**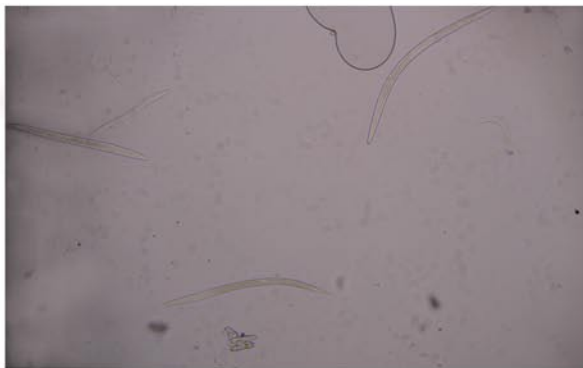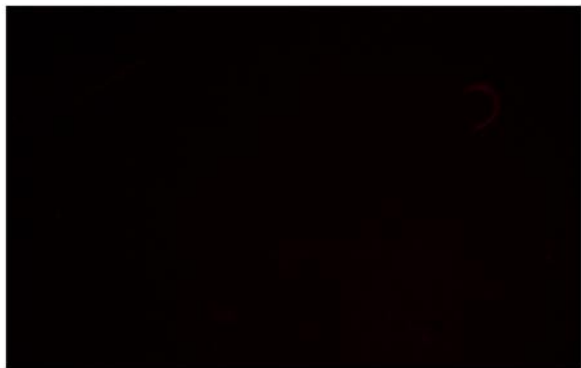**B**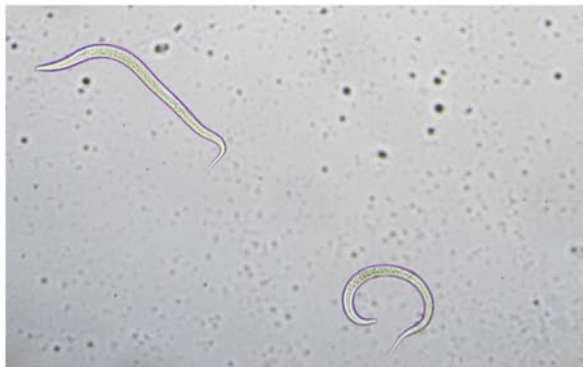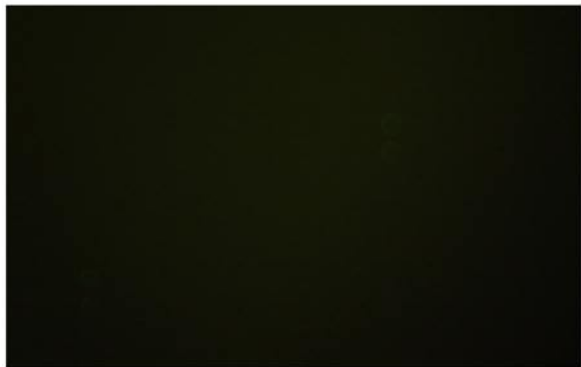

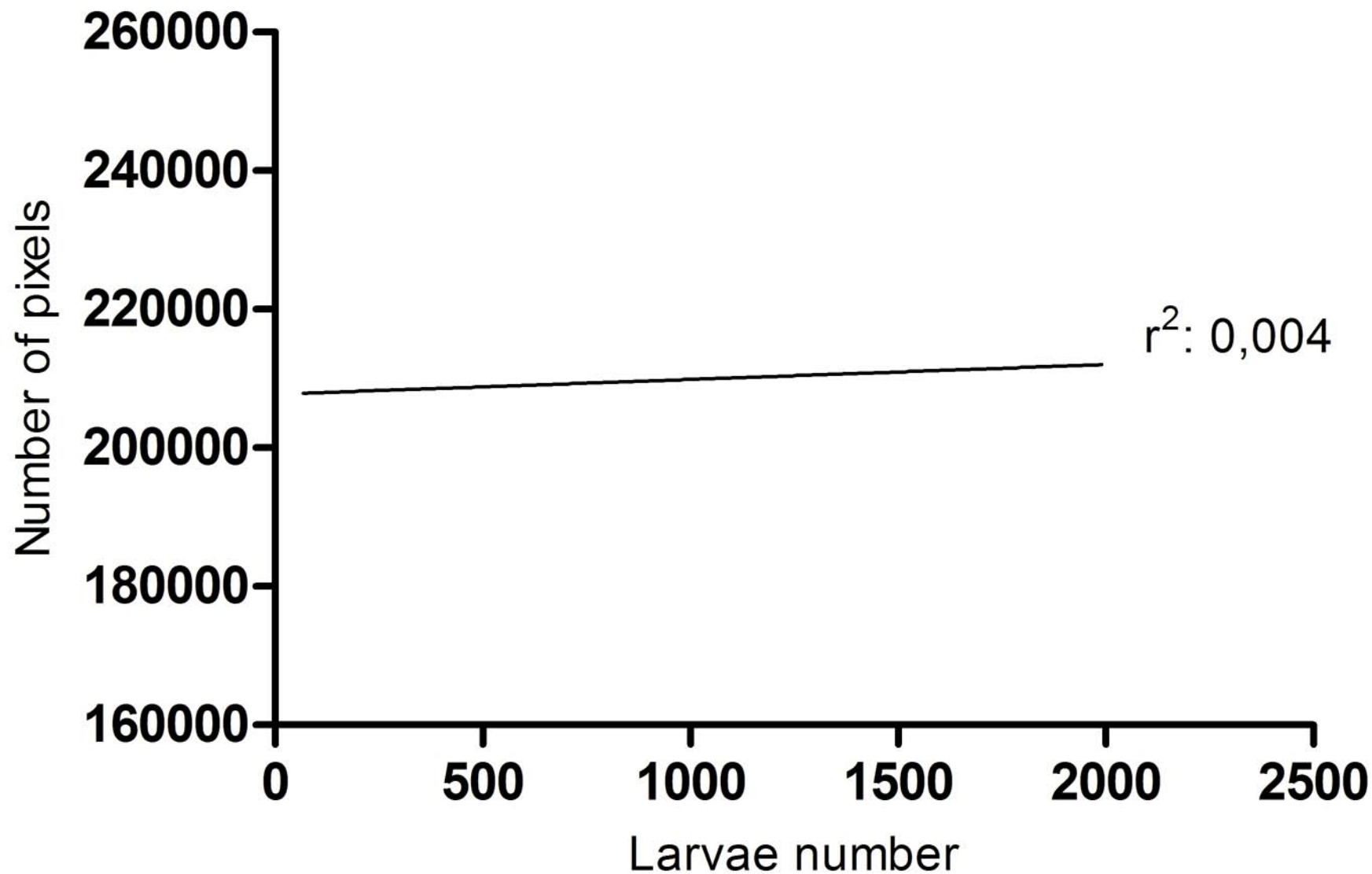

Supplement: Supplementary file 1 — The Supplementary Material demonstrate that fluorimetric markers employed in the study does not stain viable live larvae (Supplementary Figure 1) and absence of autofluorescence emitted by the larvae, demonstrate by the lack of fluorescence when dead larvae (treated with methanol) were not stained with Sytox and Propidium Iodide. Os marcadores utilizados não foram eficazes em marcar larvas viáveis (não tratadas). A fluorescência analisada referente às larvas não tratadas com metanol não apresentava proporcionalidade, de acordo os parâmetros estabelecidos. [file 879263.f1.pdf]
